# Development of a novel person-centered question prompt list to talk with your pharmacists in Japanese community pharmacies: focus group and Delphi method

**DOI:** 10.1186/s40780-025-00494-7

**Published:** 2025-10-14

**Authors:** Masayo Hayakawa, Hayato Kizaki, Yuki Yanagisawa, Nobuyuki Suzuki, Yumi Kagawa, Kyoko Sayama, Shungo Imai, Satoko Hori

**Affiliations:** 1 Division of Drug Informatics, Keio University Faculty of Pharmacy, 1-5-30 Shibakoen, Minato-ku, Tokyo, 105-8512 Japan; 2 Kan-i net, 2-18-28 Nishihokima, Adachi-ku, Tokyo, 121-0812 Japan; 3 Psychology and Behavioral Science Course, Human Science Department, Iwate Medical University Center for Liberal Arts and Science, 1-1-1 Idaidori, Yahaba-cho, Shiwa-gun, Iwate, 028-3694 Japan

**Keywords:** Health communication [MeSH], Question prompt list, Community pharmacy services [MeSH], Focus group [MeSH], Delphi method [MeSH], Patient preference [MeSH], Consumer health information [MeSH]

## Abstract

**Background:**

The concept of shifting from a patient-centered to person-centered approach in pharmacy practice has been proposed. Patient-centered care focuses on the individual patient, whereas person-centered care focuses on a holistic understanding of the individual, taking not only medications but also daily life into account. This shift requires effective communication between patients and pharmacists. Although question prompt lists (QPLs) exist for patients and doctors, the same is not available for pharmacists focused on a person-centered approach. This study aimed to develop a question prompt list to talk with your pharmacists (QPLP) focusing on a person-centered approach to facilitate communication with patients.

**Methods:**

This study aimed to develop the QPLP in three steps. In the first step, six medical pharmaceutical researchers with pharmacist qualifications and two patient researchers prepared an initial draft of the QPLP, referencing existing QPLs used by patients to prepare questions for doctors before consultation. A focus group interview was then conducted with eight patients, and a QPLP drafted. Finally, a modified Delphi method was used to evaluate and collect opinions, and the QPLP was finalized.

**Results:**

A QPLP comprising 16 questions was developed with patient participation in a three-step process. The content was categorized into five sections: “Medicines,” “How to Take Medicines,” “Daily Life,” “Treatment,” and “Consumer Health Information.” The questions covered concerns regarding medicines, difficulties in using medicines, issues in daily life during treatment, treatment-related problems, authenticity of health information, and community health and exercise information.

**Conclusions:**

In this study, we developed a novel QPLP to enhance communication between patients and pharmacists, focusing on a person-centered approach with active patient involvement. Future studies should investigate the contribution of the developed QPLP in improving patient–pharmacist communication. These questions may encourage both healthy people and patients to seek health advice from pharmacists.

**Supplementary Information:**

The online version contains supplementary material available at 10.1186/s40780-025-00494-7.

## Background

In recent years, the professional roles of pharmacists around the world have expanded into the domains of public health and primary care, highlighting the increasing importance of community pharmacies [1]. The World Health Organization and the International Pharmaceutical Federation have emphasized four key roles in the expansion of pharmacy services: assessing patient health status and needs, providing information about medicines and health-related issues, disseminating evaluated information about medicines and various aspects of self-care, and engaging in preventive care activities and services [2]. Clinical studies have reported that such pharmacist-led interventions positively influence the health of community residents [3–8]. In Japan, community pharmacies are expected to serve as the first point of contact for citizens regarding health-related concerns, and their role as a public health resource has been increasingly emphasized [9].

In recent years, a shift from a “patient-centered” to a “person-centered” approach has been proposed in pharmacy practice [10–12]. Olson and Burns explain that while these terms are often used interchangeably, they reflect distinct perspectives: patient-centered care focuses on the individual as a patient within a clinical context. In contrast, person-centered care seeks to understand the individual holistically, considering all aspects of their life to more comprehensively interpret health concerns and aspirations [13]. They further argue that pharmacists are well-positioned to integrate their clinical expertise with an individual’s lived experiences, thereby contributing to health and wellness through a co-equal partnership (to the degree desired by the person undergoing care). Given the preventive and community-oriented nature of pharmacy practice, particularly in public health contexts, a person-centered approach is increasingly warranted.

To extend pharmaceutical services and realize a person-centered approach, active patient participation is essential. While patient engagement in healthcare has been widely studied [14, 15], research that focuses specifically on patient participation in community pharmacy settings remains limited. In pharmacotherapy, active patient participation can help with the identification of medication-related problems and lead to improvements in patient comprehension of their health conditions and treatments. This requires encouraging patients to actively “ask questions,” “express concerns and worries,” and “share personal beliefs, interests, and daily life in the pharmacy.” [16, 17].

Currently, however, pharmacists rarely encourage patients to ask questions or explore their own needs [16–19]. Moreover, discrepancies exist between the information patients wish to receive—such as adverse drug reactions and drug–drug interactions—and what pharmacists actually communicate [20]. This communication gap may contribute to reduced patient satisfaction and decreased medication adherence [21, 22]. Therefore, it is important to implement person-centered communication in community pharmacies, and to enhance the quality of dialogue between pharmacists and patients [23].

Question prompt lists (QPLs) for patients, particularly in oncology, have been utilized to help patients prepare questions for doctors before their consultations [24–28]. Evidence that QPLs are useful has accumulated, and they are now routinely used in clinical practice [29]. Applications that allow patients to easily create QPLs before their consultation via websites or software programs are currently available in the United States, Australia, and other countries, and they have proven to be effective [30–32].

In the field of pharmacy, there are lists of questions that focus on appropriate use and medical safety, such as those in the 1994 “Questions To Ask About Your Medicines” campaign by the World Health Organization [33], and those devised by Svensberg et al. [34, 35]. However, no question prompt list to talk with your pharmacists (QPLP) has been developed that includes patient concerns beyond medication use, such as prevention, their life background, and challenges in treatment, which are essential elements of a person-centered approach.

Therefore, for this study, we conceived the idea of developing a person-centered QPLP to trigger communication between patients and pharmacists. In the process, we incorporated a public and patient involvement approach using focus group interviews (FGIs) with patients [36] as well as the RAND/UCLA modified Delphi method (hereafter referred to as the “modified Delphi method”) [37].

## Methods

**Fig. 1:**
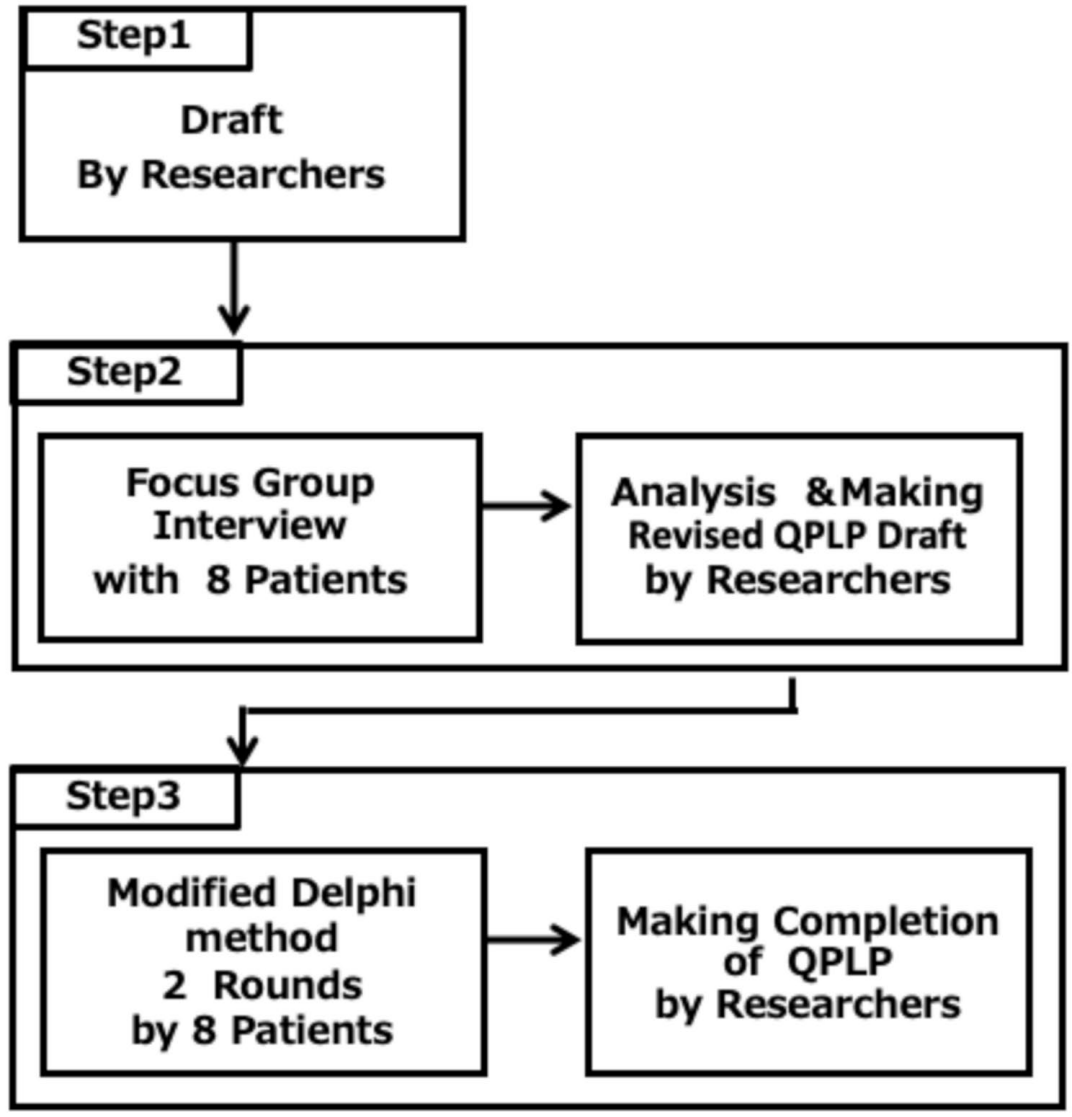
Flowchart of the development of the QPLP conducted in three steps

The QPLP was developed in three steps (Fig. 1). In the first step, we created a first draft. In the second step, FGIs were conducted with eight patients, and the QPLP was revised. In the third step, the QPLP was finalized through two rounds of evaluation using the modified Delphi method. The ethics committee of Keio University Faculty of Pharmacy (231124-1) approved the study protocol.

### Step 1: Preparation of the initial QPLP draft

We drafted the initial QPLP. The authors were six healthcare pharmacy researchers with pharmacist qualifications and two patient researchers with lived experience as patients (in contrast to pharmacist qualifications). The healthcare pharmacy researchers (MH, YY, KS, SI, SH) independently extracted questions from 119 existing QPLs [27, 38], and after discussion, the list was narrowed down to 57 questions. Through iterative discussion among the researchers, overlapping or ambiguous items were merged or excluded, thereby refining the list to 57 questions.

Two existing QPLs were used to help rephrase statements as questions to a pharmacist. One of these QPLs was the Question Builder, which was provided by Healthdirect Australia and established in August 2006 under an agreement with the Australian Council of Governments [31]. It was selected because it is still widely used and researched. The other was “For Patients and Families Preparing for Important Consultations,” which is used by cancer patients in Japan [27]. The existing QPL [33–35] used by pharmacists was not referenced because it did not include questions about anything other than medications. The patient researchers (NS, YK) participated in discussions to finalize the first draft of the QPLP. Since previous studies suggested limiting the number of questions to fewer than 20 [38] and keeping the length of the list to one page to reduce patient burden, the number of questions was further reduced to fewer than 20, and the initial draft consisted of 17 questions.

The questions were listed in order of ease of use, and were intended to be used at home or during the time they waited at the pharmacy on patients’ second or subsequent visits to the pharmacy. The aim was to promote person-centered communication by encouraging patients to ask pharmacists relevant questions and communicate their concerns, wishes, thoughts, and daily life situations, thereby enhancing communication with the pharmacists.

### Step 2: Focus group interviews and revision of the draft QPLP

The FGIs were conducted using the method of Vaughn et al. [37]. Advantages of this method include group discussions that generate stimuli about the topic and the emergence of extensive cohesive data.

#### Recruitment

Participants were recruited using patient organization mail lists and social media platforms. The inclusion criteria were as follows:

1. aged 18 or older
2. regularly visits pharmacies and has used prescription medications for more than three years
3. has experience in patient organization activities and sharing personal experiences with other patients

The third criterion was included to encourage participation by those whose experience in patient groups or other organizations allowed them to speak from a broader perspective, one that includes not only their personal experience but also the opinions of other patients.

#### Implementation of FGIs

Eight applicants responded within two weeks. They were divided into two groups of three and five members, which is a comfortable number for group discussions. The consent form was emailed in advance, explained, and collected on the day of the interviews. Separate schedules were established for each group. To ensure that participants met the criteria and did not all have the same health condition, participant background information was collected using questionnaires. The FGIs were designed to gather the opinions of participants regarding the initial QPLP draft, focusing on the prioritization of questions, as well as suggestions for additional questions not included in the draft. We used a self-developed interview guide (Supplement 1). The facilitator, experienced in FGIs and qualitative research, ensured that the participants could freely express their views.

Each FGI lasted for 1 h 45 min. The participants were three men and five women, with a median age (min, max) of 51 (30,73) years. These participants used a median of 3 [2, 11] medicines and visited their doctors approximately once a month. The participants had regularly used pharmacies for an average of 18.4 years (Table 1). Their conditions spanned a range of different areas: neurological, respiratory, cardiovascular, diabetic, gastrointestinal, psychiatric, otolaryngological, oncological, ophthalmological, and gynecological (Table 1).

**Table 1:** Participant characteristics in the focus group interview and modified Delphi methods (*N* = 8)

**Age (in years)**	**Number of Participants**
18–29 30–39 40–49 50–59 60–69 70–79 Over 80	0 1 3 2 1 1 0
**Sex**	**Number of Participants**
Male Female	3 5
**Number of pharmacy visits in the last 12 months**	**Number of Participants**
0–10 11–20 21–30	5 1 2
**Duration of regular pharmacy use (in years)**	**Number of Participants**
0–10 11–20 21–30 31–40	3 3 0 2

The questions were revised and adjusted through the FGIs, resulting in a revised draft of 16 questions.

#### Analysis of FGI data and preparation of the revised QPLP

Audio data from the FGIs were transcribed verbatim, using anonymized personally identifiable information that was then used for analysis. From the data, opinions were extracted for each QPLP question. These comments were reviewed and discussed by all researchers. The adoption or rejection of each question as well as its wording were revised. For questions related to “treatment,” we were mindful not to exceed the scope of the pharmacist’s role by inappropriately acting as a medical practitioner.

### Step 3: Evaluation and finalization of the QPLP using the modified Delphi method

The modified Delphi method [37] was employed to both evaluate the QPLP and collect patient opinions. The method is a structured consensus-building technique that collects expert opinions through multiple rounds with controlled feedback. It allows anonymous participation, reduces bias from dominant individuals or groups, and enhances the convergence of opinions in fewer rounds by incorporating feedback and discussion among experts.

#### Implementation of the modified Delphi method

We conducted two rounds of evaluations and collected additional opinions using the modified Delphi method. Both rounds were conducted via email with the same participants from the FGIs using self-developed questionnaires (Supplements 2, 3). In Round 1, the participants rated if each of the 16 questions should be included in the QPLP using a five-point scale (Strongly Agree, Agree, Neutral, Disagree, Strongly Disagree) and provided reasons for their ratings. The feedback from Round 1 was aggregated, and all data were shared with the participants without modification. Three questions proposed in the first round were added, and 19 questions were evaluated in the second round. In the second round, participants re-evaluated the questions using the same five-point rating scale. To avoid bias, communication related to the modified Delphi method was managed by individuals not involved in the survey or analysis, with the researchers receiving only anonymized data.

#### Analysis of the data from the modified Delphi method and preparation of the final QPLP

The researchers confirmed that opinions had been aggregated through the two-round Delphi method and discussed the aggregated Round 2 data, providing their opinions on each question. As the QPLP is intended only as an example list to facilitate patients in asking questions, it was considered important to ensure that it caused no harm. Therefore, consensus was defined such that any item that even one participant rated as “disagree” or “strongly disagree” was deleted. This approach reflects the consensus-building process of the Delphi method while prioritizing patient safety and acceptability.

For the remaining questions, the text was adjusted based on the opinions of the researchers.

## Results

The QPLP was developed using the following three steps.

### Step 1: Creation of the initial QPLP draft

We finalized the initial draft with 17 questions grouped into five categories in Step 1.

The patient researchers suggested that pharmacists should communicate the following two messages to patients when asking them to use the QPLP: “Please ask if your medicine fits your lifestyle,” and “Please talk about any concerns regarding your medicine and treatment; let’s think together with the pharmacist.” They also suggested that these messages should be prepared as explanatory documents accompanying the list. To make the list easier to read, the questions were divided into five categories based on their contents.

### Step 2: Focus group interviews and preparation of the revised QPLP draft

**Fig. 2:**
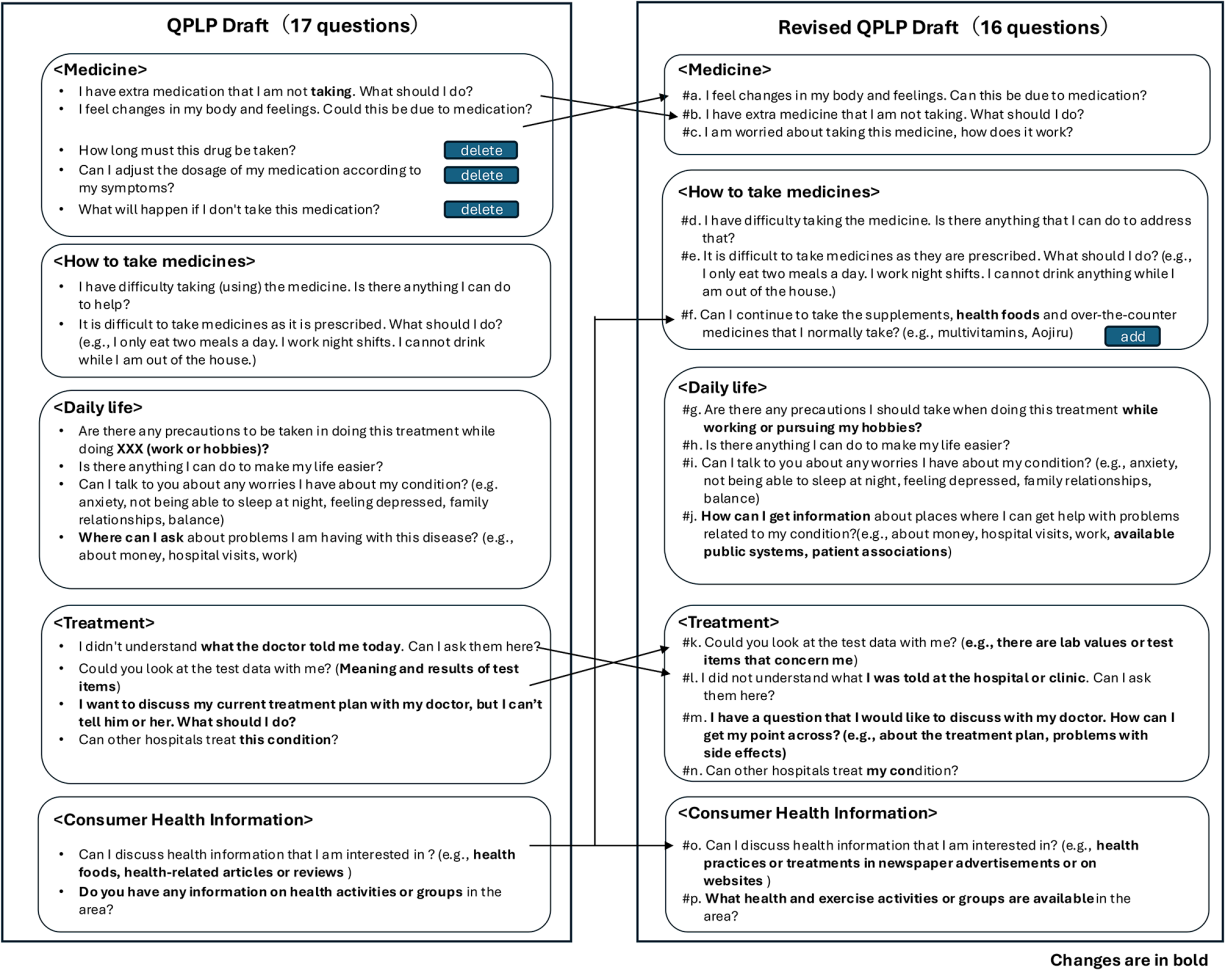
Results of the FGI

In Step 2, 17 questions (carried over from Step 1) were evaluated in the FGIs. Based on participant feedback, three questions were removed, one question was divided into two, and one additional question was added; accordingly, the revised draft comprised 16 questions (Fig. 2). Three questions that were deemed difficult or inappropriate for the pharmacists to answer accurately were removed: “*How long must this drug be taken?*” “*Can I adjust the dosage of my medication according to my symptoms?*” and “*What will happen if I don’t take this medication?*” This is because, in Japan, pharmacists do not have prescriptive authority and cannot adjust the dosage of drugs, and not all medical information is shared. In contrast, questions were added to address issues that doctors might not always explain, such as “*How does this medication work? Is it safe to take?*” Furthermore, many participants wished to ask pharmacists questions about supplements and health foods, reflected by the question, “*Can I continue to take the supplements*, *health foods*, *and over-the-counter medicines that I normally take? (e.g.*, *multivitamins*, *Aojiru)*,” which was added later.

There was much discussion about two questions: “*This has been revised to align with the wording in Fig. 2 for consistency. I didn’t understand what that the doctor told me today. Can I ask them here?*” and “*I want to discuss my current treatment plan with my doctor*, *but I can’t. What should I do?*” One participant mentioned that he had heard of a case in which felt unable to put his question to a doctor, so he asked a pharmacist instead. These questions concerned the patient’s desire to understand the doctor’s explanation. In addition, patients sought advice on how they should approach their doctors to facilitate discussions. The question “*I want to discuss my current treatment plan with my doctor*, *but I can’t tell him or her. What should I do?*” was changed to an open-ended question, “*I have a question that I would like to discuss with my doctor. How can I get my point across? (e.g. about the treatment plan*, *problems with side effects)*”since “treatment plan” refers to only one example.

The order of the questions was also adjusted to make it easier for patients to ask questions. For example, some patients said, “*I have medicine that I am not taking. What should I do?*” This was not an easy question for them to ask because it was essentially a confession that they were not taking their medicine. Therefore, we moved that question to the second part of the list.

### Step 3: Finalization of the QPLP using the modified Delphi method

The same eight individuals who participated in the FGIs also participated in the modified Delphi method. Sixteen questions were evaluated in Round 1.

Two questions (#r and #s in Table 2) were deleted, since they elicited either “Disagree” or “Strongly Disagree” responses in Round 2. Two other questions (#h and #i in Tabel2) were combined into one question: “*Is there anything I can do to make my daily life easier?” [Question #i]* was combined with “*Are there any precautions [that] I should take while I take this treatment? [Question #h]*. In the end, 16 questions remained.

**Table 2:** Results of the modified Delphi method

Delphi No	Revised Draft QPLP in Step 2 (17 questions) + questions added after Delphi Round 2 (3 questions)	Strongly agree	Agree	Neither	Disagree	Strongly disagree	Changes following Delphi results
		Upper row: Round 1 (%) Lower row: Round 2 (%)					
**<Medicine>**							
#a	I feel changes in my body and feelings. Can this be due to medicine?	75.0	25.0				
		75.0	25.0				
#b	I have extra medicine that I am not taking. What should I do?	87.5			12.5		
		75.0	12.5	12.5			
#c	I am worried about taking this medicine, how does it work?	62.5	37.5				modified wording
		87.5	12.5				
**<How to take medicines>**							
#q	What should I do if I forget to take my medicine?	-	-	-	-	-	
	[Added by R2]	100.0					
#d	I have difficulty taking the medicine. Is there anything that I can do to address that?	62.5	37.5				
		75.0	25.0				
#e	It is difficult to take medicines as they are prescribed. What should I do? (e.g., For example, I only eat two meals a day. I work night shifts. I cannot drink anything while I am out of the house.)	75.0	12.5	12.5			modified wording
		87.5	12.5				
#f	Can I continue to take the supplements, health foods and over-the-counter medicines that I normally take? (e.g., multivitamins, Aojiru)	87.5	12.5				modified wording
		100.0					
**<Daily life>**							
#g	Are there any precautions I should take when doing this treatment while working or pursuing my hobbies?	62.5	37.5				Combined with #i and modified wording
		62.5	37.5				
#h	Is there anything I can do to make my life easier?	37.5	37.5	12.5		12.5	Combined with #h
		50.0	37.5	12.5			
#i	Can I talk to you about any worries I have about my condition? (e.g., anxiety, not being able to sleep at night, feeling depressed, family relationships, balance)	62.5	12.5	25.0			
		75.0	12.5	12.5			
#j	How can I get information about places where I can get help with problems related to my condition? (e.g., about money, hospital visits, work, available programs, patient associations)	62.5	25.0	12.5			modified wording
		37.5	50.0	12.5			
**<Treatment>**							
#k	Could you look at the test data with me? (e.g., there are lab values or test items that concern me)	50.0	37.5	12.5			
		75.0	25.0				
#l	I did not understand what I was told at the hospital or clinic. Can I ask them here?	62.5	37.5				
		75.0	25.0				
#m	I have a question that I would like to discuss with my doctor. How can I get my point across? (e.g., about the treatment plan, problems with side effects)	62.5	37.5				
		50.0	50.0				
#n	Can other hospitals treat my condition?	62.5	25.0	12.5			
		87.5		12.5			
**<Consumer Health Information>**							
#o	Can I discuss health information that I am interested in? (e.g., health practices or treatments in newspaper advertisements or on websites)	75.0	25.0				modified wording
		62.5	37.5				
#p	What health and exercise activities or groups are available in the area?	25.0	50.0	12.5	12.5		modified wording
		50.0	12.5	37.5			
#r	Can you recommend a nearby restaurant (or sweet shop)?	-	-	-	-	-	
	[Added by R2]	37.5	37.5	12.5		12.5	**deleted**
#s	Self-introductions, weather topics, mood questions	-	-	-	-	-	
	[Added by R2]	37.5	25.0	12.5	12.5	12.5	**deleted**

Rating on a five-point scale (Strongly Agree, Agree, Neutral, Disagree, Strongly Disagree)

There were two questions that all respondents strongly agreed with: “*Can I continue to take the supplements*, *health foods and over-the-counter medicines I normally take? (e.g.*, *multivitamins*, *Aojiru) [Question #g]*”” and “*What should I do if I forget to take my medicine? [Question #d]* ”. *[Question #d]* was one of the additional questions proposed in Round 1. Based on the opinions from the Delphi method (Table 3), a third question was revised as follows: “*I am worried about taking this medication*, *how does it work? [Question #c]*” was changed to “*I am worried about taking this medicine. Can you tell me more about how it works* *and what its side effects are?” [Question #3]*. The revised questions were made applicable to patients receiving not only initial prescriptions but also continued prescriptions.

**Table 3:** Questionnaires and opinions modified as a result of the modified Delphi method

Delphi No	Final QPLP No	Revised QPLP draft	Modified questionnaire.	Opinions/Modifications
#c	#3	I am worried about taking this medicine, how does it work?	I am worried about taking this medication. Can you tell me more about **how it works and what its side effects are**?	- The two main questions to ask are: how does it work, and what are the side effects? This question should apply to patients presenting with either initial or continuing prescriptions.
#e	#6	It is difficult to take medications as they are prescribed. What should I do? (e.g., I only eat two meals a day. I work night shifts. I cannot drink anything while I am out of the house.)	It is difficult **to follow the timing of taking my medicines.** What should I do? **(e.g.**, **I work nights or go out a lot.)**	- The examples in this question are important because there are many interpretations. -Only eating two meals a day is an insufficient explanation. - The expression ‘as it is prescribed’ is difficult to understand.
#f	#7	Can I continue to take the supplements, health foods and over-the-counter medicines I normally take? (e.g., multivitamins, Aojiru )	Can I take any supplements, health foods, or over-the-counter medicines **in addition to these medicines**? (e.g., multivitamins, **nutritional drinks**, aojiru)	- I think this is a good question that takes advantage of the pharmacist’s strengths regarding drug combinations. - I feel that many people take nutritional drinks along with their medicines, so it would be good to include “nutritional drinks.” However, it seems that some drugs should not be taken with these drinks. - Including Aojiru, a green vegetable drink, in this question is not ideal because of its narrow scope as a term. However, it is not a product name, and therefore should be included. Drug interactions are common with Aojiru.
#g, h	#8	#h Are there any precautions I should take when doing this treatment while working or pursuing my hobbies? #i Is there anything I can do to make my life easier? #i was combined with #h	Is there anything I can do to make my days easier **while continuing the treatment? (e.g.**, **I would like to work and pursue my hobbies.)**	- How about integrating 8 and 9? - “I feel that many people with serious illnesses do not believe they can pursue the ‘treatment while working or doing hobbies’.” - How about changing “to make it easier to live day to day” to “to make it easier” (not adopted, as side effects would be assumed) - Opinion from a pharmacist in response to the Delphi results. Need to ask about work and hobbies to demonstrate expertise. Can talk to them about their lifestyle, such as when to take a laxative before going out, or if it is better to take it 30 min before the pain is likely to occur.
#j	#10	How can I get information about places where I can get help with problems related to my condition? (e.g., about money, hospital visits, work, available programs, patient associations)	Can you tell me where toask for help with my illness or problems in my life? (e.g., **living expense support programs**, **continuing to work**, **referrals to patient associations**)	- The text was concise, and the examples were organized. - It is a question that requires preparation on the part of the respondent.
#o	#15	**Can I discuss health information that I am interested in?** (e.g., health practices or treatments in newspaper advertisements or on websites)	**I have health information I am interested in. May I ask you about it?** (e.g., health practices or treatments in newspaper advertisements or on websites)	
#p	#16	What health and exercise activities or groups are available in the area?	Can you tell me about any health or exercise-related activities or groups in this community (**neighborhood association**, **local government**, etc.)?	- Added examples

The wording of the seven questions was changed after being reviewed by the researchers based on feedback from the Delphi method. The five categories into which the questions were divided were “Medicine,” “How to Take Medicine,” “Daily Life,” “Treatment,” and “Consumer Health Information.” The questions were aimed at addressing concerns about medicines (difficulties in using medicines, problems with daily treatment, and concerns about treatment), the authenticity of health information, and community health and exercise information (Table 4 and Supplement 4). The patients commented that the list made them realize that they could talk to pharmacists about these issues.

**Table 4:** Question prompt list to talk with your pharmacists (QPLP) developed in this study

**<Medicine>**	
#1	I feel changes in my body and feelings. Can this be due to medicine?
#2	I have extra medicine that I am not taking. What should I do?
#3	I am worried about taking this medication. Can you tell me more about how it works and what its side effects are?
**<How to take medicines>**	
#4	What should I do if I forget to take my medicine?
#5	I have difficulty taking medicine. Is there anything that I can do to address that?
#6	It is difficult to follow the timing of taking medicines. What should I do? (e.g., I work nights or go out a lot.)
#7	Can I take supplements, health foods, or over-the-counter medicines in addition to these medicines? (e.g., multivitamins, nutritional drinks, and aojiru).
**<Daily life>**	
#8	Is there anything I can do to make my days easier while continuing the treatment? (e.g., I would like to work and pursue my hobbies).
#9	Can I talk to you about my worries about my condition? (e.g., anxiety, inability to sleep at night, feeling depressed, family relationships, and balance)
#10	Can you tell me where to ask for help with my illness or problems in my life? (e.g., living expense support programs, continuing to work, and referrals to patient associations).
**<Treatment>**	
#11	Could you examine the test data with me? (e.g., there are lab values or test items that concern me.)
#12	I did not understand what I was told at the hospital or clinic. Can I ask them here?
#13	I would like to discuss a question with my doctor. How can I get my point across? (e.g., regarding the treatment plan and problems with side effects)
#14	Can other hospitals treat my condition?
**<Consumer Health Information>**	
#15	I have health information I am interested in. May I ask you about it? (e.g., health practices or treatments in newspaper advertisements or websites)
#16	Can you tell me about any health or exercise-related activities or groups in this community (neighborhood associations, local governments, etc.)?

## Discussion

This study developed a QPLP with the aim of encouraging patients to talk with their pharmacists questions. This was based on a person-centered approach and implemented in collaboration with patients to improve patient–pharmacist communication. The initial draft of the QPLP was created by researchers, including healthcare providers and patients. Questions were then added, deleted, or modified by the patients following FGIs and using a modified Delphi method, which resulted in a final list of 16 questions. These were evaluated and categorized into five groups: “Medicines,” “How to Take Medicines”, “Daily Life,” “Treatment” and “Consumer Health Information.”

In contrast, existing QPLs for pharmacists primarily contained items related to the categories of “Medicines” and “How to Take Medicines,” such as “Have any medicines been added/removed?” or “How should I use the medicine?” [33–35]. However, the QPLP developed in this study expanded beyond these conventional domains and included questions categorized under “Daily Life,” “Treatment,” and “Consumer Health Information.” This indicates that the QPLP not only supports discussions about pharmacological aspects but also addresses patients’ broader health-related concerns and everyday challenges, thereby facilitating communication based on a person-centered approach. The questions in the “Medicines” and “How to Take Medicine” categories were designed to focus on common patient concerns rather than on what the pharmacists typically want patients to ask about or be aware of regarding proper medicine take. For example, the wording of the questions was designed to begin with the patient’s perspective, such as “*I am worried about taking this medicine. Can you tell me more about what its side effects are?” [Question #3]* rather than a question phrased from a healthcare professional’s perspective, such as “*What are the most typical side effects of the medicine?*” [33] This approach ensured that the questions were in tune with patient feelings and experiences. Furthermore, “*I have extra medicine that I am not taking. What should I do?” [Question #2]*, which was moved down in the order of questions, was set as the first question by the pharmacist researchers during the drafting phase as they thought that it was a question that patients would be comfortable asking. However, the participants commented that it was difficult for them to ask such questions, as it would imply admitting that they were not adhering to their medications. This highlights the importance of a person-centered approach in the QPLP development process.

The questions in the “Daily Life,” “Treatment,” and “Consumer Health Information” categories included questions relevant to both patients and healthy people, representing a broadening of the list used previously [33, 34].

For the patients, these questions addressed areas in which they may have felt hesitant to ask questions of their pharmacists. For pharmacists, these questions were critical, as they could prompt conversations that lead to medical support tailored to the patient’s lifestyle.

Questions #7, #15, and #16 are questions that community members who do not regularly visit pharmacies also want to talk, but do not know whom to talk. Pharmacists have the potential to advise these patients. In particular, question #15 (*I have health information I am interested in. May I ask you about it?* [e.g., health practices or treatments in newspaper advertisements or websites]), which relates to the reliability of information abundantly available on the internet, was problematic [39] because they have no one to ask for advice. Question #16 was about local health support information; community pharmacies are increasingly contributing to health promotion, and are expected to collaborate with other healthcare agencies [40]. These questions may encourage both patients and the healthy population to seek health advice from pharmacists, which could lead to pharmacies providing more community-based, person-centered services and contribute positively to the field of primary care [41].

Questions #10 and #16 relate to information on consultation services and local health activities, which are thought to be related to the role of social prescribing in connecting people to local resources [42]. Relevant materials connecting people to community resources should be prepared for community pharmacies.

In order for the QPLP to be utilized in the future, a statement of purpose should be prepared and shown to users before they see the list. Educational materials are requested for pharmacists because doctors ask for information and training on how to use the QPLs and respond to questions [38, 43].

In addition to using the QPLP, improving patient–pharmacist communication requires multiple approaches, including enhancing the pharmacy environment. According to “Patient Participation in Pharmacy Care Model” by Qudah et al., factors like the presence of companions, pharmacy layout, type of pharmacy, and external factors such as waiting time can hinder communication between pharmacists and patients [44].

Through this study, we aimed to develop a QPLP with active patient participation and input. In the future, it will be important to evaluate whether the QPLP effectively improves patient–pharmacist communication once it is put into practice, and to establish a continuous cycle of feedback and improvement. Furthermore, as the expansion of professional roles in public health and primary care in pharmacies is anticipated, public perception of the role of community pharmacies in public health needs to be assessed [45].

### Strengths, limitations and future scope

This study has a number of limitations. First, there is a lack of generalizability regarding the results of the FGI and Delphi methods. Patients who regularly used community pharmacies were asked to participate while those who rarely visited pharmacies or received home care were excluded. However, the participants were individuals who had participated in activities such as patient support groups where they had shared information about their health experiences with other patients. They had various health conditions, and we believe that their experiences in these activities allowed them to reflect on patient opinions from a broad perspective. Second, the number of participants in this study was limited (*n* = 8). While we did attempt to overcome participant bias by recruiting patients of various ages and with diverse medical histories, resulting in improved dynamics and diverse ideas, studies with larger numbers of participants should be encouraged. Third, the study population was limited to patients with chronic diseases. Although the questions classified under the “Health Information” category of the developed QPLP is not restricted to this group and may also be relevant for healthy individuals and those without chronic conditions. As person-centered care emphasizes involvement across the entire health process, including the pre-disease stage, future research that incorporates the perspectives of healthy individuals and patients without chronic diseases may be valuable to improve the generalizability and clinical applicability of the QPLP.

## Conclusions

The QPLP developed in this study was designed with patient participation to enhance communication between patient and pharmacist. It was created within the concept of patient-centered care, which focuses on the individual as a patient, plus the concept of person-centered care, which considers not only medications but also day-to-day life, with an eye toward understanding the individual holistically.

The QPLP has the potential to improve patient satisfaction, adherence to medication, and overall health outcomes by encouraging patients to ask questions and express concerns. These questions may encourage both healthy people and patients to seek health advice from pharmacists. This could lead to pharmacies providing more community-based, person-centered services and contributing positively to the field of primary care. However, further studies are needed to evaluate the effectiveness of QPLP in real-world settings and its impact on patient–pharmacist communication.

## Supplementary Information

Below is the link to the electronic supplementary material.

Supplement 1: Focus Group Interview Guide

Supplement 2: The questionnaire of the Delphi Method Round 1

Supplement 3: The questionnaire of the Delphi Method Round 2

Supplement 4: QPLP Japanese version

## Data Availability

No datasets were generated or analysed during the current study.

## Abbreviations

QPLP: Question prompt list to talk with your pharmacists
QPL: Question prompt list
FGI: Focus group interview
