# Supplementary material for: Development of a novel person-centered question prompt list to talk with your pharmacists in Japanese community pharmacies: focus group and Delphi method
Source: J Pharm Health Care Sci. 2025 Oct 14;11:87. doi: 10.1186/s40780-025-00494-7 (PMC12522932; doi:10.1186/s40780-025-00494-7)
Supplement: Supplementary file 1 — Supplement 1: Focus Group Interview Guide [file 40780_2025_494_MOESM1_ESM.docx]

Supplement 1.　 Focus Group Interview Guide

1. Participants introduce themselves, the group interview process is explained, and consent is obtained via the consent form.
2. Each participant reviews the draft of the QPLP and summarizes their opinions.
3. Participants are asked to share their opinions in turn.
4. Which questions in the draft QPLP do you think would make it easier for you to speak with the pharmacist?
5. Which questions in the draft QPLP would you feel hesitant to ask?
6. What questions are missing from the draft QPLP that you think should be included?
7. Allow participants to share additional opinions freely.

Would you like to add anything to your earlier statement after hearing each other's thoughts?

1. Thank the participants and handle any necessary administrative communication.

| **The draft the QPLP**  **<Medicine>**   - I have extra medication that I am not taking. What should I do? - I feel changes in my body and feelings. Could this be due to medication? - How long must this drug be used? - Can I adjust the dosage of my medication according to my symptoms? - What will happen if I don't take this medication?   **<How to take/use medicines>**   - I have difficulty taking the medicine. Is there anything I can do to help? - It is difficult to take medicines as it is prescribed. What should I do? (For example, I only eat two meals a day. I work night shifts. I cannot drink anything while I am out of the house.)   **<Daily life>**   - Are there any precautions to be taken with this treatment while doing XXX (work or hobbies)? - Is there anything I can do to make my life easier? - Can I talk to you about any worries I have about my condition? (e.g. anxiety, not being able to sleep at night, feeling depressed, family relationships, balance) - Where can I ask about problems I am having with this disease? (For example, about money, hospital visits, work)   **<Treatment>**   - I didn't understand what the doctor told me today. Can I ask about it here? - Could you look at the test data with me? (Meaning and results of test items) - I want to discuss my current treatment plan with my doctor, but I can't to tell him or her. What should I do? - Can other hospitals treat this condition?   **<Consumer Health Information>**   - Can I discuss health information that I am interested in? (e.g., health foods, health-related articles or reviews.) - Do you have any information on health activities or groups in the area? |
| --- |
