# Supplementary material for: Development of a novel person-centered question prompt list to talk with your pharmacists in Japanese community pharmacies: focus group and Delphi method
Source: J Pharm Health Care Sci. 2025 Oct 14;11:87. doi: 10.1186/s40780-025-00494-7 (PMC12522932; doi:10.1186/s40780-025-00494-7)
Supplement: Supplementary file 3 — Supplement 3: The questionnaire of the Delphi Method Round 2 [file 40780_2025_494_MOESM3_ESM.docx]

Supplement 3. The questionnaire of the Delphi Method Round 2

The Revised Question Prompt List for Pharmacist (QPLP; 16 questions) with 3 additional questions

- On a scale of 1 to 5, please rate each of the following questions in terms of how useful you think they would be for improving communication with pharmacists:

[1 = strongly agree, 2 = agree, 3 = neutral, 4 = disagree, 5 = strongly disagree].

- If you believe any wording should be changed, please provide a suggested revision. You are also welcome to share any additional comments or suggestions.

The ratings and comments submitted will be shared with other respondents, without revealing their names.

| Categories | No | Questions (The text has not been changed from the previous version.) | Ratings:  Please enter a number from 1 to 5 | Previous Rating Average  Average of the 5-point scale, based on 1 (strongly agree) to 5 (strongly disagree), as rated by 8 respondents  A smaller value indicates a higher likelihood of the question being considered important for inclusion in the list*. |
| --- | --- | --- | --- | --- |
| Medicine | a | I feel changes in my body and feelings. Can this be due to medication? |  | 1.3 |
|  | b | I have extra medicine that I am not taking. What should I do? |  | 1.4 |
|  | c | I am worried about taking this medicine, how does it work? |  | 1.4 |
| How to take medicines | d | I have difficulty taking the medicine. Is there anything that I can do to address that? |  | 1.4 |
|  | e | It is difficult to take medicines as they are prescribed. What should I do? (e.g., I only eat two meals a day. I work night shifts. I cannot drink anything while I am out of the house.) |  | 1.4 |
|  | f | Can I continue to take the supplements, health foods and over-the-counter medicines that I normally take? (e.g. multivitamins, Aojiru) |  | 1.1 |
| Daily life | g | Are there any precautions I should take when doing this treatment while working or pursuing my hobbies? |  | 1.4 |
|  | h | Is there anything I can do to make my life easier? |  | 2.1 |
|  | i | Can I talk to you about any worries I have about my condition? (e.g. , anxiety, not being able to sleep at night, feeling depressed, family relationships, balance) |  | 1.6 |
|  | j | How can I get information about places where I can get for help with problems related to my condition? (e.g., about money, hospital visits, work, available public systems, patient associations.) |  | 1.5 |
| Treatment | k | Could you look at the test data with me? (e.g., there are lab values or test items that concern me.) |  | 1.6 |
|  | l | I did not understand what I was told at the hospital or clinic. Can I ask them here? |  | 1.4 |
|  | m | I have a question that I would like to discuss with my doctor. How can I get my point across? (e.g. about the treatment plan, problems with side effects) |  | 1.4 |
|  | n | Can other hospitals treat my condition? |  | 1.5 |
| Consumer Health Information | o | Can I discuss health information that I am interested in? (e.g. health practices or treatments in newspaper advertisements or on websites) |  | 1.3 |
|  | p | What health and exercise activities or groups are available in the area? |  | 2.1 |
| Added | q | What should I do if I forget to take my medicine? |  |  |
| Added | r | Can you recommend a nearby restaurant (or sweet shop)? |  |  |
| Added | s | Self-introductions, weather topics, mood questions |  |  |

Other comments (optional)

|  |
| --- |

* For this dataset, it is customary to use the median; however, using the median resulted in a value of “1” for most items, making it difficult to distinguish between them. The final decisions, however, were not based on these values.
