# Supplementary material for: Development of a novel person-centered question prompt list to talk with your pharmacists in Japanese community pharmacies: focus group and Delphi method
Source: J Pharm Health Care Sci. 2025 Oct 14;11:87. doi: 10.1186/s40780-025-00494-7 (PMC12522932; doi:10.1186/s40780-025-00494-7)
Supplement: Supplementary file 4 — Supplement 4: QPLP Japanese version [file 40780_2025_494_MOESM4_ESM.docx]

Supplement 4.　QPLP　Japanese version　 日本語版

＜薬について＞

1. からだや気持ちに変化を感じています。薬の影響が考えられますか。
2. 使っていない薬が余っています。どうすればいいですか。
3. この薬を使うことに不安があります。効き方や副作用などを詳しく教えてくれますか。

＜薬の飲みかた/使いかた＞

1. 薬を飲み忘れたとき（使い忘れたとき）はどうすればいいですか。
2. 薬が飲みにくい（使いにくい）です。何かいい方法はありますか。
3. 薬を飲む（使う）タイミングを守るのが難しいです。どうすればいいですか。
   （たとえば、夜勤や外出が多いなど）
4. この薬と一緒にサプリメント、健康食品、市販薬を使ってもいいですか。
   （たとえば、マルチビタミン、栄養ドリンク、青汁）

＜生活＞

1. 治療を続けながら日々を過ごしやすくするために、何かできることはありますか。
   （たとえば、仕事や趣味もしていきたい）
2. 病気にともなう心配ごとについて相談できますか。
   （たとえば、不安で眠れない、気分が落ち込む、家族への説明の仕方）
3. 病気や生活で困っていることを聞ける相談先を教えてくれますか。

（たとえば、生活費支援制度、仕事の継続、患者会の紹介）

＜治療＞

1. 検査データを一緒に見てもらえませんか。
   （たとえば、気になる検査値や検査項目など）
2. 病院やクリニックで言われてよく分からなかったことがあります。ここでお聞きしてもいいですか。
3. 医師に相談したいことがあります。どう言えば伝わりやすいですか。
   （たとえば、治療方針、副作用での困りごと）
4. 他の病院でも私の病気の治療を受けられますか。

＜健康情報＞

1. 気になっている健康情報があります。お聞きしてもいいですか。
   （たとえば、新聞広告やウェブサイトにある健康法や治療法）
2. この地域（町内会、自治体など）で行われている健康や運動に関する活動やそのグループを教えてくれますか。
